# Inhibition of glycosphingolipid synthesis reverses skin inflammation and hair loss in ApoE−/− mice fed western diet

**DOI:** 10.1038/s41598-018-28663-9

**Published:** 2018-07-30

**Authors:** Djahida Bedja, Wenwen Yan, Viren Lad, Domenica Iocco, Nickash Sivakumar, Veera Venkata Ratnam Bandaru, Subroto Chatterjee

**Affiliations:** 1 Johns Hopkins University School of Medicine, Baltimore, Maryland USA; 2 Medical Toxicology Research Division, Biochemistry and Physiology Branch, Analytics, United States Army Medical Research Institute of Chemical Defense (USAMRICD), 8350 Ricketts Point Road, Aberdeen Proving Ground, Edgewood, Maryland 21010-5400 USA; 3 Present Address: Department of Cardiology, 3-Tonji Hospital affiliated to Tonji University, Shanghai, 200065 China

## Abstract

Sphingolipids have been accorded numerous biological functions however, the effects of feeding a western diet (diet rich in cholesterol and fat) on skin phenotypes, and color is not known. Here, we observed that chronic high-fat and high-cholesterol diet intake in a mouse model of atherosclerosis (ApoE−/−) decreases the level of ceramides and glucosylceramide. At the expense of increased levels of lactosylceramide due to an increase in the expression of lactosylceramide synthase (GalT-V). This is accompanied with neutrophil infiltration into dermis, and enrichment of tumor necrosis factor-stimulated gene-6 (TSG-6) protein. This causes skin inflammation, hair discoloration and loss, in ApoE−/− mice. Conversely, inhibition of glycosphingolipid synthesis, by D-threo-1-phenyl-2-decanoylamino-3-morpholino-1-propanol (D-PDMP), unbound or encapsulated in a biodegradable polymer (BPD) reversed these phenotypes. Thus, inhibition of glycosphingolipid synthesis represents a unique therapeutic approach relevant to human skin and hair Biology.

## Introduction

Glycosphingolipids (GSLs) are integral components of all cell membranes, and affect numerous biological functions^1^. Glycosphingolipids are synthesized by the sequential transfer of monosaccharides such as glucose, from the nucleotide sugar, UDP-glucose, to ceramide to form glucosylceramide (GlcCer)^2^. Analogously, the subsequent transfer of galactose from UDP-Gal to GlcCer, via the activity of a galactosyltransferase, produces lactosylceramide (LacCer). Such GSLs are then assembled into very low-density lipoproteins (VLDLs), in the liver, and secreted into circulating blood. Herein, VLDL is modified to cholesterol and GSL-rich low-density lipoprotein (LDL) particles, for delivery to peripheral tissues, including the skin, the most predominant tissue in mammals. The uppermost layer of the skin, the stratum corneum, is made up of enucleate corneocytes, which are surrounded by extracellular lipids, to form multi-lamellar structures. These lipids thus impart an overwhelmingly impenetrable barrier function to the stratum corneum^3^. Thus, glycosphingolipids constitute a major species of lipids in human skin, skin fibroblasts, and keratinocytes^4–6^. Prior studies showed that normal human skin fibroblasts can not only synthesize GSLs, but also take up GSLs from LDLs, via an LDR receptor-dependent pathway^7^. LDL receptor expression has also been correlated with keratinocyte differentiation^8,9^ and barrier function^3^. Keratinocytes also regulate the activity of melanocytes cells responsible for pigmentation of the eye, skin, and hair, thus providing protection from the external environment, including ultraviolet rays^10–14^. These pioneering studies led us to hypothesize that alterations in skin glycosphingolipid homeostasis may affect dermal biology.

Dermatitis is a common skin disorder effecting 1 to 21% of mouse population in research laboratories and facilities^15–17^, leading to unnecessary euthanasia^18^ and thus loss of important data. Dermatitis was reported to occur in the face, head and neck area in the C57BL/6 mice and mice with the same genetic background^15–18^. Scratching, the affected area due to pruritus and fights, increases the severity of ulceration^18^. Other risk factors that may predispose mouse to this disorder including abnormal grooming behavior before the onset of lesion, high fat diet, gender differences as well as aging^15,17,19–25^. The apoE−/− mice was generated by gene inactivating apolipoprotein E (ApoE)^26,27^. It regulates lipid homeostasis and functions as a ligand for receptors that transport and clears its lipids such as chylomicrons, cholesterol, low and very low density lipoprotein (LDL, VLDL) remnants from different tissue and cell^26,27^. There are three homozygous phenotype including: Apo E2, ApoE3 and ApoE4 alleles present in men^27–29^. The Apo E2 and E4 variants are associated with significantly higher risk of cardiovascular events and Alzheimer’s disease^27–29^.

On a chow diet, the cholesterol level of ApoE−/− mice range of ~500 mg/dl^30^. However, feeding a western diet markedly increases cholesterol levels^31^.Lack of ApoE gene in these homozygous mice also impedes with the dietary absorption and biliary excretion of cholesterol^32^. A review on diet and murine atherosclerosis^33^ reported that when fed a western diet the ApoE−/− mice had lipid deposits in liver, colon, lung and skin^34^. This is the first time we report these results of skin whitening in Apo E−/− mice fed high fat and high cholesterol

Nonetheless, the mechanism of this disorder/dermatitis is still unknown and treatment with triple anti-biotic ointment, sulfadiazine, and toes trimming as well as others methods may not be effective but are variable in effectiveness^15,17,18,35^.

The disturbance of the Notch pathway^36–42^ by inactivating of Notch1, Notch2, or RBP-Jk in melanocyte lineage using Tyr::Cre transgenic mice resulted in gene dosage-dependent hair graying, with pronounced effect in mice deficient in both Notch 1 and Notch 2^43–45^

Most recent studies on mice deficient in stem cell factor in the transcription factor called krox20 lineage that exhibit hair graying exposed the identities of hair matrix progenitors which regulate hair growth and pigmentation^36^. And creating a stem cell factor-dependent niche for follicular melanocytes^46^

Herein, we used a mouse model of atherosclerosis, ApoE−/−, to determine the effects of consumption of a high fat and high cholesterol diet on skin GSL composition, and skin inflammation, involving inflammatory cells (e.g., neutrophils), and tumor necrosis factor-alpha (TNF-alpha)-stimulated gene-6 (TSG-6). As TSG-6 binds to the extracellular matrix component hyaluronan^47^, and is associated with wounding and inflammatory conditions, including neutrophil migration^48^ we tested our hypothesis that inhibition of GSL synthesis could interfere with inflammation of the skin, with subsequent effects on hair coloration and hair loss, in ApoE−/− atherosclerotic mice fed a western diet.

## Results

### Feeding a western diet leads to skin dys-homeostasis, preventable by the glycosphingolipid inhibitor BPD

**Figure 1:**
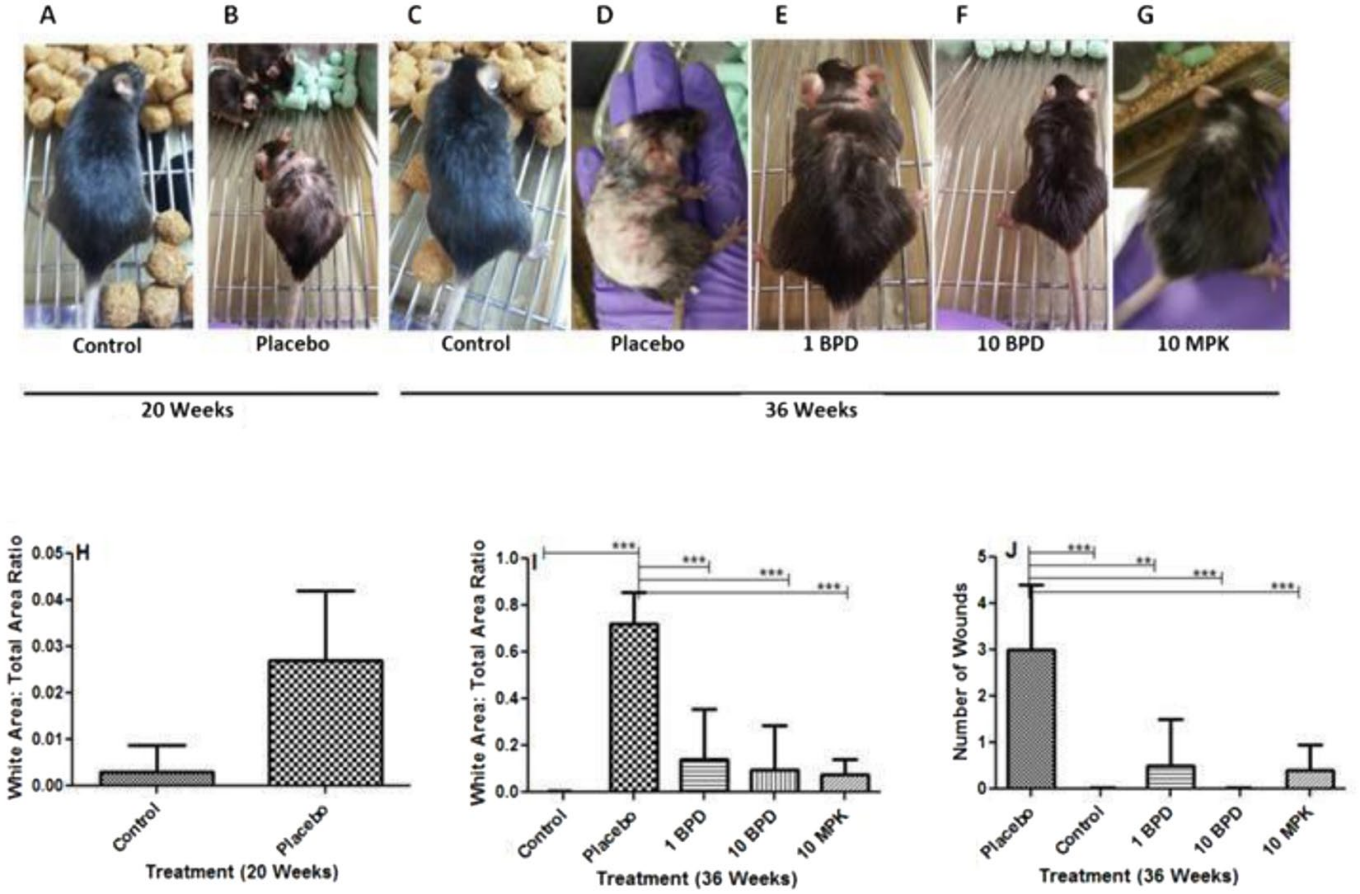
Phenotypes of Apo E−/− mice skin and hair fed with and without western diet. (**A**) represent mice fed normal chow diet. (**B**,**H**) ApoE−/− mice fed a western diet from 12–20 weeks with moderately increased hair whitening, loss of hair, skin inflammation and lesion formation. **(D**,**I**) shows extensive loss of hair, skin lesion when the western diet was continued to 36 weeks of age, compared to ApoE−/− mice fed chow for 36 weeks **(C**,**I)**. (**E**,**F)** mice fed a combination of western diet and 1 mpk BPD **(E)** and 10 mpk BPD **(F)**, from 20 weeks to 36 weeks, with ameliorated hair discoloration, hair loss, and skin inflammation 1 G. Mice treated with 10 mpk unbound D-PDMP and fed western diet. This treatment also reduced the occurrence of these pathologies **(G**,**I**). Note treatment with 1 mpk BPD was as efficient as 10 mpk of native /un-encapsulated D-PDMP in regard to hair discoloration and hair loss **(I)**. (N = 5 p < 0.05). Quantitative analysis shows 75% of skin area had hair loss, discoloration and inflammation **(I**) in placebo mice. Treatment with BPD or D-PDMP interfered with this phenotype in dose-dependent. And 1 mpk of BPD was as effective (87%) as 10 mpk of D-PDMP (83%) in wound healing **(I,J)**. A nonparametric one –way ANOVA using the Bonferroni’s comparison test was performed. *p < 0.05, **p < 0.001, ***p < 0.001; n = 3–5.

ApoE−/− mice were fed normal chow or a western diet, continuously from the age of 12 to 20 weeks. Next, mice were fed 1 mg/kg (mpk) 5, 10 mpk of BPD (encapsulated form of the GSL synthesis inhibitor, PDMP), and 10 mpk (unbound) D-PDMP, daily, by oral gavage, from the age of 20 to 36 weeks (time of atherosclerosis onset), with continuation of the same diet. We observed that compared to mice fed normal chow **(**Fig. 1A), ApoE−/− mice fed a western diet from 12–20 weeks of age had modestly increased hair whitening, loss of hair, and skin lesion formation **(**Fig 1B,H**)**, compared to Apo−/− mice fed a normal chow diet. When the western diet was continued to 36 weeks of age, we noted a marked loss of hair, extensive whitening of the skin (Fig. 1D,I**)**, compared to ApoE−/− mice fed normal mice chow for 36 weeks **(**Fig. 1C,I**)**. Moreover, feeding 1 mpk BPD **(**Fig. 1E**)** and 10 mpk BPD **(**Fig. 1F**)**, concurrent with the western diet, from 20 weeks to 36 weeks, ameliorated hair discoloration, hair loss, and skin inflammation **(**Fig. 1E–G**)**. Analogously, treatment with 10 mpk unbound D-PDMP also interfered with these pathologies **(**Fig. 1G,I**)**. In addition, ApoE−/− mice fed a western diet for 36 weeks had numerous small and large inflammatory wounds/skin lesions **(**Fig. 1D,J**)**, compared to ApoE−/−mice fed normal chow **(**Fig. 1C**)**. However, treatment with 1 mpk and 10 mpk of BPD markedly promoted wound healing. Treatment during 20–36 weeks of age with 1 mpk BPD was as efficient as 10 mpk of native /un-encapsulated D-PDMP **(**Fig. 1K) (N = 5 p < 0.05).

Quantitative analysis of whitening of skin was done by scanning the area from the neck to the back of mice **(**Fig. 1I**)**. We observed that placebo mice had ~75% whitening of hair/hair loss, and treatment with BPD or D-PDMP interfered with this phenotype. We also observed that feeding a high fat and cholesterol caused wounding of the skin **(**Fig. 1J**)**. Treatment with BPD dose-dependently interfered with wounding of the skin. And treatment with1 mpk of BPD was as effective (87%) as 10 mpk of D-PDMP (83%) in wound healing.

### Neutrophil infiltration and TSG-6 expression in skin is ameliorated by Treatment with BPD

**Figure 2:**
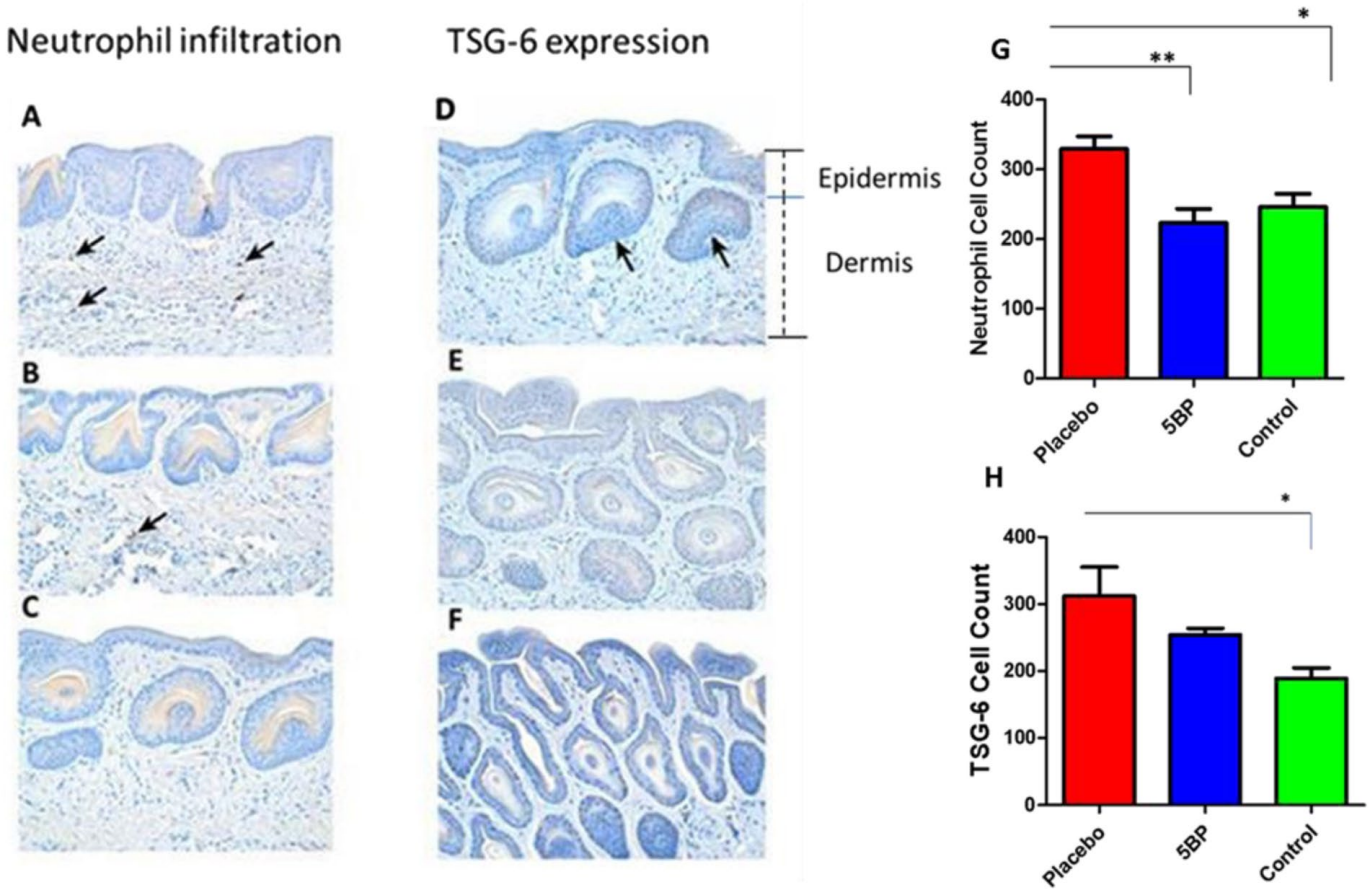
Neutrophil infiltration and TSG-6 Expression Representative thin sections of skin stained with an antibody against neutrophils (CD166), and counterstained with hematoxylin-eosin. Feeding western diet resulted in the infiltration of neutrophils, into various skin dermal areas (**A**, see arrows) as compared to controls **(C)**. In contrast, BPD treatment significantly reduced the number of infiltrating neutrophils **(B)**. Immunostained skin section **(D)**, with an antibody against the TSG-6 protein, finding that show positive reaction in mice were fed a western diet **(D)**, vs. control mice on normal chow diet **(F)**. In contrast, mouse skin sections did not react positively to this antibody, following treatment with BPD **(E)**. Demonstrating BPD’s high efficacy, in reversing skin neutrophil migration, via TSG-6 down-regulation and inhibition of skin lesion formation/inflammation. (Magnification (20x).Treatment normalized the neutrophil count in the skin (**G**). Similarly, treatment reduced the TSG-6 count /TSG-6 expression (**H**). A nonparametric one –way ANOA using the Bonferroni’s multiple comparison test was performed. *p < 0.05, **p < 0.001, ***p < 0.001; n = 5–6.

When representative thin sections of skin were stained with an antibody against neutrophils (CD166), and counterstained with hematoxylin-eosin, we observed that feeding a western diet resulted in the infiltration of neutrophils, into various skin dermal areas **(**Fig. 2A, see arrows**)** as compared to controls **(**Fig. 2C**)**. In contrast, BPD treatment significantly reduced the number of infiltrating neutrophils **(**Fig. 2B**)**. Since tumor necrosis factor, a pro-inflammatory cytokine, is known to stimulate several genes, including tumor necrosis factor-stimulated gene-6 (TSG6), we also immunostained skin sections with an antibody against the TSG-6 protein, finding that skin sections reacted positively when mice were fed a western diet **(**Fig. 2D), vs. control mice (i.e., normal chow) **(**Fig. 2F**)**. In contrast, mouse skin sections did not react positively to this antibody, following treatment with BPD **(**Fig. 2E**)**. The neutrophil count and TSG-6 values in placebo, 5BP treated and control mice values demonstrate BPD’s high efficacy, in reversing skin neutrophil migration/count **(**Fig. 2G**)**, and TSG-6 **(**Fig. 2H**)** down-regulation and inhibition of skin lesion formation/inflammation.

### Biopolymer-encapsulated D-PDMP is superior to unbound D-PDMP, in restoring hair homeostasis

**Figure 3:**
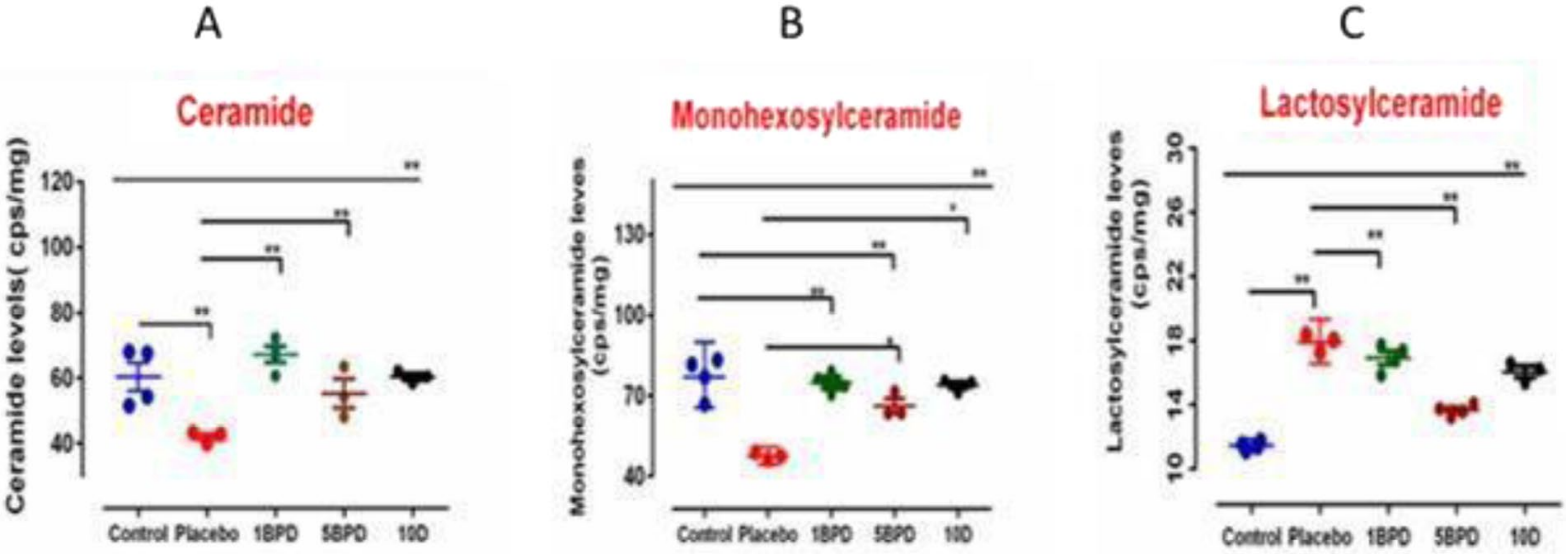
Total Ceramide, Monohexosylceramide and Lactosylceramide levels in skin samples. Skin tissue were subject to total lipid extraction and MS-MS analysis in mice fed chow (blue/control)a western diet(red/ placebo),western diet + 1 mpk of biopolymer –encapsulated D-PDMP(green/1BPD) daily by oral gavage from age 20 weeks to 36 weeks, western diet +10 mpk-BPD(10BPD/pink) and western diet + 10 mpk of D-PDMP(10D/). As compared to normal chow, mice fed western diet alone(red) decreased the total ceramide and monoglycosylceramide mass, whereas the level of lactosylceramide increased. Treatment with the un-encapsulated (D-PDMP) and biopolymer–encapsulated D-PDMP (BPD) increased total ceramide and glucosylceramide levels, in a dose-dependent, to near-normal levels. Both BPD and D-PDMP treatment decreased total lactosylceramide levels in the skin **(C)**. A nonparametric one –way ANOA using the Bonferroni’s multiple comparison test was performed. *p < 0.05, **p < 0.001, ***p < 0.001; n = 3–5.

MS-MS analysis revealed that mice fed a western diet had relatively low total ceramide levels of d18:1/16:0, d18:1/18:0, d18:1/20:0, d18:1/22:0, d18:1/24:0, d18:1/26:0, d18:1/18:1, d18:1/20:1, d18:1/22:1, d18:1/24:1, d18:1/26:1, d18:0/18:0, d18:0/22:0 and d18:0/24:0. **(**Fig. 3A**)**, compared to mice fed regular chow **(**Fig. 3A**)**. Similarly, total mohexosylceramide levels of d18:1/18:1, d18:1/20:1, d18:1/24:1, d18:1/26:1, d18:1/16:0, d18:1/18:0, d18:1/20:0, d18:1/22:0, d18:1/24:0), d18:1/26:0, d18:0/16:0, d18:0/18:0, d18:0/20:0, d18:0/22:0 and d18:0/24:0 were lower in mice fed a western diet **(**Fig. 3B**)**. In contrast, total lactosylceramide levels of d18:1/16:0, d18:1/18:0, d18:1/20:0, d18:1/22:0, d18:1/24:0, d18:0/16:0, d18:0/18:0 and d18:0/20:0 were significantly increased in mice fed a western diet, compared to controls **(**Fig. 3C**)**. Upon feeding BPD, we observed that total ceramide and glucosylceramide levels increased, in a dose-dependent manner, to near-normal levels. Similarly, treatment with the un-encapsulated inhibitor, D-PDMP, albeit at a significantly higher dose than the biopolymer–encapsulated D-PDMP, also increased ceramide and glucosylceramide levels. Conversely, treatment with either BPD or D-PDMP decreased total lactosylceramide levels in the skin **(**Fig. 3C).

Detailed mass spectrometric analysis of control mice skin ceramides showed that d18:/24:0, d18:/26:1, d18:/16:1, d18:1/24:1, d18:1/22:1, d18:1/18:0, and d18:1/20:0 were the predominant fatty acid molecular species (in the hydrophobic “tails” of ceramides), in a descending order **(**Fig. S1A-G**)**. Feeding a western diet decreased, by ~3-fold, the levels of almost all fatty acid molecular species of ceramide, except for C18:1/24:0, which did not change significantly **(**Fig. S1E**)**. Treatment with 1 mpk BPD, or 10 mpk D-PDMP, however, increased them to normal levels. However, treatment with 10 mpk D-PDMP did not reverse the loss of d18:1/26:1 ceramide, nor did increasing the dose of BPD from 1 to 5 mpk, affect levels of skin ceramides, and thus, the negative consequences of aging, including pigmentation and hair loss.

Following D-PDMP treatment, we noted distinct differences in the fatty acid molecular composition of skin ceramides, and their glycosylated derivatives. For example, whereas fatty acid molecular species d18:1/16:0, d18:1/22:0, and d18:1/26:0 were absent from skin ceramides, these three species were present in skin monohexosylceramide. Moreover, the level of d18:1/16:0 monohexosylceramide was modestly increased in mice fed the western diet **(**Fig. S2**)**, but remained unchanged with BPD treatment. Also the level of monohexosylceramide containing d18:1/18:0 and d18:1/22:0 remained unchanged, with or without a western diet, or following treatment **(**Fig. S2**)**. Levels of skin monohexosylceramide, having 18:1/24:0 tails, decreased by ~1.5-fold, upon western diet feeding. This level was reversed, however, upon treatment **(**Fig. S2E), and 1 mpk D-PDMP was as efficacious as treatment with 10 mpk BPD. However, such treatment raised the level of d18:1/24:1, and 1 mpk BPD was superior to treatment with 10 mpk D-PDMP. Without treatment, the levels of skin monohexosylceramide containing d18:1/24:1 **(**Fig. S2F**)** and d18:1/26:0, decreased by ~ 3-fold, in mice fed a western diet. Treatment with 1 mpk BPD, but not 10 mpk D-PDMP, completely restored skin monohexosylceramide levels containing d18;1/24;1 **(**Fig. S2F**)** fatty acids, which were similar to monohexosylceramides containing the d18:1/26:0 fatty acid **(**Fig. S2G).

The most dramatic observation in the skin of 36 weeks old mice, fed a western diet, was a 3 –fold increase in lactosylceramide having a d18:1/16:0 fatty acid, compared to control mice on a normal diet **(**Fig. S3A**)**. Moreover, this effect was modestly decreased upon BPD or D-PDMP treatment **(**Fig. S3A**)**. Conversely, levels of lactosylceramide having d18:1/18:0 decreased ~2-fold in mice fed a western diet, compared to controls, and this was also reversed upon drug treatment **(**Fig. S3B**)**. Analogously, levels of lactosylceramide having d18:1/22:0 **(**Fig. S3C**)** and d18:1/24:1 (Fig. SBD) molecular species were also increased 1.5-fold and 2-fold, respectively, in the skin of mice fed a western diet. These levels were also restored to normal, upon treatment with 1 mpk BPD or 10 mpk D-PDMP **(**Fig. S3C,D).

### Treatment with BPD decreases the mass of B1, 4-Galactosyltransferase (GalT-V)

**Figure 4:**
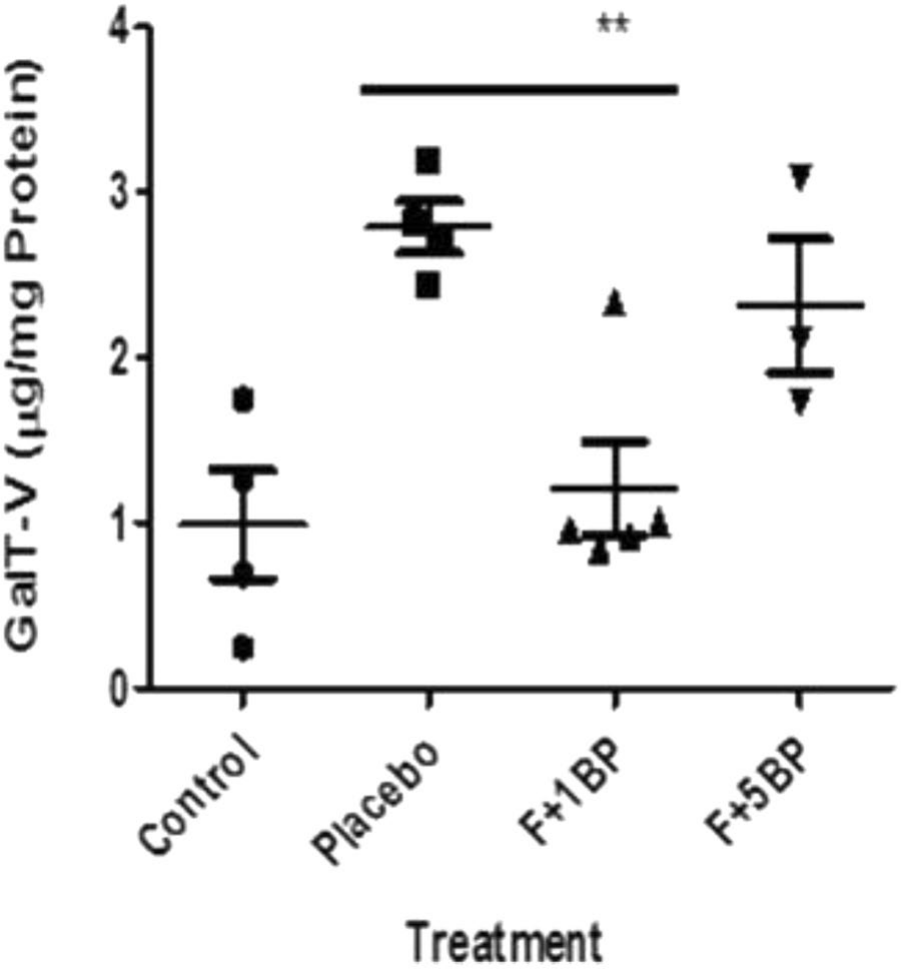
Increased lactosylceramide synthase (GalT-V) level in APOE−/− mice is reversed upon treatment with D-PDMP/ BPD. Skin tissue from ApoE−/− mice fed normal chow or western diet with and without treatment with D-PDMP and BPD were homogenized in RIPA buffer, centrifuged and the supernatant used as a source of lactosylceramide synthase (GalT-V). The mass of GalT-V was measured using an ELISA assay and antibody raised against this antigen. We observed increased mass of GalT-V in placebo mice compared to control mice. Conversely, treatment with BPD decreased the mass of GalT-V, with 1BPD being more effective than 5BPD in skin tissue. A nonparametric one –way ANOA using the Bonferroni’s multiple comparison p test was performed. *p < 0.05, **p < 0.001, ***p < 0.001; n = 3–5.

ELISA assays in skin tissue revealed increased mass of GalT-V, upon feeding a western diet to ApoE−/− mice **(**Fig. 4**)**, as compared to controls. However, treatment with BPD dose-dependently decreased the mass of GalT-V, with 1 mpk of BPD being as effective as 5-mpk D-PDMP, in decreasing GalT-V levels in skin tissue.

## Discussion

**Figure 5:**
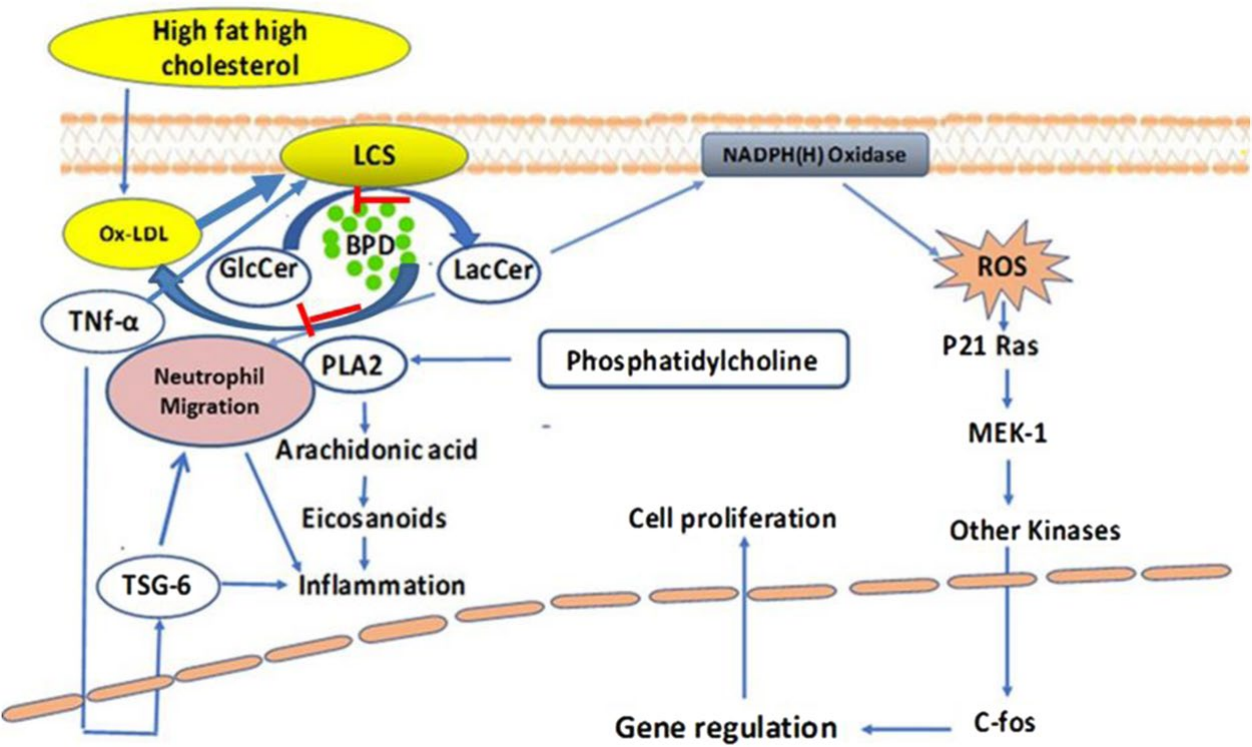
Mechanism of action of lactosylceramide in skin inflammation and restoration by the use of D-PDMP. Feeding a western diet raises the level of oxidized phospholipid which activates Lactosylceramide synthase (GalT-V) to generate lactosylceramide which produces reactive oxygen species-a pro-oxidant environment. Lactosylceramide also reacts with infiltrating neutrophils to activate phospholipase A-2, thus releasing arachidonic acid, eicosanoids and contributing to inflammation. This cycle of events can be broken by the judicious use of D-PDMP/BPD.

In this study, we examined the effects of the glycosphingolipid synthesis inhibitor D-threo-1-phenyl-2-decanoylamino-3-morpholino-1-propanol (D-PDMP), with (“BPD”) and without encapsulation in a biodegradable polymer. Our previous study has shown that D-PDMP inhibits the activity of glucosylceramde synthase as well as lactosylceramide synthase^49^. Both free and encapsulated D-PDMP affected three adverse consequences of aging on hair biology, hair loss, skin inflammation, and pigment loss. The key observations emerging from this study were: (1) feeding a western diet to ApoE−/− atherosclerotic mice exerts a time-dependent loss in hair color, loss of hair, and skin inflammation/lesion formation, accompanied by neutrophil infiltration and TSG-6 expression; (2) these phenotypic changes were accompanied by significantly decreased skin ceramides and monoglycosylceramides, and increased levels of lactosylceramide; (3) treatment, with either unconjugated/native D-PDMP or biopolymer-encapsulated D-PDMP (BPD), not only prevented loss of hair coloration and skin inflammation, but also reversed these phenotypes to near-normal; and (4) BPD, compared to free D-PDMP, is the superior, more potent, and effective mode of drug delivery. A hypothetical model explaining biochemical/molecular mechanisms, by which lactosylceramide plays a role in skin inflammation, and its restoration by the use of D-PDMP/BPD, is presented in Fig. 5.

Although pioneering studies by Elias *et al*., have shown an important role for long-chain fatty acid-containing ceramides in maintaining skin hydration, the effects of feeding a western diet, to our knowledge, has not been previously investigated^3,4^. The ApoE−/− mouse is a transgenic model of atherosclerosis, having significant defects in the homeostasis of cholesterol and other lipids^50,51^. These mice have thick black hair when fed a normal mouse chow diet. However, upon feeding a high fat and high cholesterol diet, the hair color turns from black to gray to white and finally, is shed- nearly completely lost by 36 weeks of age. We would note that such progression is identical to that which occurs in normal human aging. This is also accompanied by the appearance of numerous hematosed skin lesions. Our data suggest that inflammation of the skin is accompanied by the enrichment of the tumor necrosis factor-inducible factor, TSG-6 **(**Fig. 2B,H) as well as infiltration of neutrophils **(**Fig. 2A,G**)**; these phenoypes were inhibited by BPD treatment. TSG-6 gene expression is induced by TNF-α and IL-1^52,53^. Studies show that TSG-6 has a hyaluronan-binding domain, qualifying this protein to be member of the hyaluronan-binding family of proteins associated with matrix stability, cell migration, and inflammation^52,53^. Thus, increased expression of TSG-6 in the skin of western diet-fed mice may well associate with inflammation and migration of circulatory neutrophils into the skin **(**Fig. 2**)**, being similar to levels expressed in a human male in his late 50s^54,55^. Clearly, further studies are warranted to elaborate our understanding of these observations.

Previous studies have shown that neutrophils are highly enriched in lactosylceramide^56^. And, treatment of these cells with lactosylceramide recruited PKC-alpha/E^57–59^ and activated phospholipase A-2, releasing arachidonic acid from phosphatidylcholine^57^ **(**Fig. 5**)**. This release activated a signaling pathway stimulating human neutrophils to upregulate Mac-(CD11b), and generate reactive oxygen metabolites that adhere to the endothelium, via increased expression of the cell adhesion molecule platelet-endothelial cell adhesion molecule (PECAM-1)^57,58,60^. The role of Src kinase, a member of the large family of tyrosine kinases mediating LacCer –induced superoxide generation of human neutrophils, has been documented as well^59,61^. *In vitro* studies in human arterial endothelial cells and monocytes showed that LacCer could not only serve as a surrogate to TNF-α and VEGF-meditated ICAM-1, PECAM-1 expression respectively, but also independently stimulates the expression of those cell adhesion proteins. These findings implicate these cell adhesion proteins in trans-endothelial migration of monocytes and neutrophils, considered a “hallmark” of the pathogenesis of inflammation^60^. Conversely, treatment with D-PDMP or use of siRNA to ablate β-1,4 galactosyltransferase-V (GalT-V) gene expression in human arterial endothelial cells blunted the adverse effects of TNF-α and VEGF signaling^1^. These findings were also corroborated using rat primary astrocytes, wherein LacCer was also implicated in the regulation of TNF-α -induced proliferation^1^. And, *in vivo* studies, using a rat model of spinal cord injury, which increased TNF-α and IL-1β mRNA expression in the lesion epicenter, associated with white matter vacuolization and loss of myelin and locomotor function suggestive of relevance to neurodegenerative disorders. These processes were obstructed by inhibition of LacCer synthesis via D-PDMP treatment^30^. Moreover, increased levels of GlcCer and LacCer have been previously reported in human plaques^1^ and more recently, plaque inflammation^1^. The latter studies showed that GlcCer and LacCer levels correlated well with several pro-inflammatory cytokines (e.g. interleukin-6, macrophage inflammatory protein (MIP-1B), and monocyte chemoattractant protein-1 (MCP-1)^1^. Taken together, these *in vitro* and *in vivo* studies may suggest a central role for LacCer, and LacCer synthase, in inflammation, by way of neutrophil infiltration and via TSG-6 expression. Consequently, our report, that feeding a western diet to ApoE−/− mice dramatically affects lost hair coloration, hair loss, and skin inflammation, processes reversible by inhibiting LacCer synthesis, further bolster this tenet, offering a novel therapeutic application for LacCer synthase inhibitors, in hair pigmentation and mitigating skin inflammation.

Previous studies of LacCer fatty acid molecular species in human atherosclerotic plaques, as well as calcified plaques, suggest a marked enrichment of d18:1/24:0, d18:1/24:1LacCer^1^. When human proximal tubular cells were fed LacCer, derived from human atherosclerotic plaques, a marked increase in cell proliferation was observed, as compared to cells fed LacCer containing d16:0/16:1 or d18:0/d18:1 fatty acid molecular species^1^. Subsequent studies revealed that, mole per mole, the longer chain fatty acid “tails” of LacCer generate more reactive oxygen species than shorter chain ones^58–60^. In the present study, we also observed the presence of LacCer, having d18:1/16:0, 18:1/24:0 and d18:1/24:1 fatty acid molecular species in ApoE−/− mouse skin. Further, feeding those mice a western diet markedly increased the level of these specific LacCer molecular species. Conversely, feeding a western diet plus D-PDMP/BPD decreased the levels of d18:1/24:0 ceramide and d18:1/26:1 ceramide. Our previous study with aortic tissue from ApoE−/− mice revealed that feeding a western diet increased the activity of GlcCer synthase and LacCer synthase, significantly increasing levels of both GSL endproducts^25^. In comparison, in mouse skin, the level of GlcCer did not change significantly upon feeding a western diet or D-PDMP or BPD **(**Fig. 3**)**. Thus, one plausible mechanistic explanation for increased LacCer levels in skin, from western diet-fed mice, could be increased synthesis of LacCer, at the expense of depleting the pool of ceramide. Conversely, treatment with D-PDMP or BPD inhibited LacCer synthase mass accumulation in skin, similar to the effects of these inhibitors in aortic tissue^25^, thus restoring ceramide to near-normal levels. We did not examine whether more complex GSL are affected in inflamed skin. However, this possibility exists and could be the subject of additional studies in the near future.

Our quantitative analysis was restricted to the following molecular species of skin GSL: Total ceramides of d18:1/16:0, d18:1/18:0, d18:1/20:0, d18:1/22:0, d18:1/24:0, d18:1/26:0, d18:1/18:1, d18:1/20:1, d18:1/22:1, d18:1/24:1, d18:1/26:1, d18:0/18:0, d18:0/22:0 and d18:0/24:0. Total monohexosylceramides of d18:1/18:1, d18:1/20:1, d18:1/24:1, d18:1/26:1, d18:1/16:0, d18:1/18:0, d18:1/20:0, d18:1/22:0, d18:1/24:0), d18:1/26:0, d18:0/16:0, d18:0/18:0, d18:0/20:0, d18:0/22:0 and d18:0/24:0. Total lactosylceramides of d18:1/16:0, d18:1/18:0, d18:1/20:0, d18:1/22:0, d18:1/24:0, d18:0/16:0, d18:0/18:0 and d18:0/20:0. However, acyl-GlcCer is also present in skin tissue. The latter group of GSL were not analyzed in our study.

We have conducted additional q-RT-PCR studies to determine the mRNA mass of these two glycosyltransferases, namely glucosylceramide synthase (UCGC) and LacCer synthase (GalT-V) in skin tissue. We observed no statistical difference in the mRNA mass (data not shown) between placebo ApoE−/− mice and ApoE−/−mice fed D-PDMP. Thus D-PDMP treatment did not alter transcriptional regulation of LacCer synthase and GlcCer synthase in skin tissue in this report as well as in liver and brain tissue in these mice and other mouse models of human disease (unpublished). However, we have already provided the protein mass of GalT-V determined using an ELISA assay **(**Fig. 4**)** showing a decrease upon feeding D-PDMP. We speculate that this could be due to an increase in the catabolism of GalT-V in D-PDMP fed mice compared to placebo mice. Clearly further studies are warranted to explore this tenet.

We have previously shown that in ApoE−/− mice fed a high fat and cholesterol diet, treatment with D-PMDP dose –dependently decreased the activity of glucosylceramide synthase and Lactosylceramide synthase in liver tissue from the same group of mice which provided us the skin tissue used in the present study^25^. Hence we did not conduct studies to measure the activity of these two enzymes in the skin.

Since treatment with D-PDMP did not decrease sphingomyelin levels, it is unlikely that increased ceramide levels in mice fed D-PDMP or BPD was due to enhanced catabolism of this phospholipid, via activation of one or more sphingomyelinases^49^.

Previously we found that the encapsulation of D-PDMP within a biopolymer, consisting of polyethylene glycol and sebacic acid, increased gastrointestinal absorption of D-PDMP, and increased its longevity in ApoE−/− mice from less than 1 hr, for the unconjugated D-PDMP, to ~48 hours^52–62^. Consequently, 1 mpk of BPD was equally efficacious as 10 mpk of free D-PDMP in decreasing LacCer levels in aortic tissue in ApoE−/− mice^37^. The preparation of polymer, nano particle formulation and encapsulation of D-PDMP to yield BPD has been described previously^52,53^. Studies on the fate of orally fed BPD has been monitored by planar Gamma scintigraphy as well its pharmaco-kinetics in several tissues in normal mice (C57Bl-6) has been described by us previously^60,63^. The present study extends this previous observation to the skin tissue. Thus, the important findings from this study were that biopolymer encapsulation is a superior mode of D-PDMP delivery than the unconjugated or native D-PDMP, in mitigating skin inflammation. Although there is ample evidence that D-PDMP can decrease the activity of GlcCer synthase and LacCer synthase^49^, we observed that treatment with BPD or D-PDMP did not markedly decrease the mass of GlcCer **(**Fig. 3B**)**. This could be explained on the basis that in skin BPD/D-PDMP is relatively more effective inhibitor of LacCer synthase as compared to other tissues. It is also possible that D-PDMP may be converted to its keto amine derivative or other derivatives, which may increase GlcCer synthesis. Alternatively, GlcCer is not catabolized properly in D-PDMP/BPD fed mice. Little is known about the functionality of proteins and enzymes when exposed to biopolymers (BP). At present we can speculate that feeding 1 mg/Kg body weight of BP-encapsulated D-PDMP provides proteins such as glucosylceramide synthase and LC synthase in a sol-gel trapping environment relatively more stable (compared to 5BP). Thus allowing these enzymes to fold into specific conformations and improved functionality.

In conclusion, to our best knowledge this is the first report wherein we show that feeding a western diet to ApoE−/− mice could have profound effects on the skin such as whitening inflammation, hair discoloration, and hair loss. Moreover, these deleterious events could be inhibited by the judicious use of a biopolymer-encapsulated inhibitor of glycosphingolipid synthesis, BPD that is relatively more efficacious than the native/unconjugated D-PMDP. Since treatment with D-PDMP also lowers the serum levels of cholesterol, oxidized LDL and triglycerides^25,62^ in addition to decreasing the level of GSL, the reversal in skin and hair pathology reported here may well be due to a combined effect on these lipids. We expect that the observations we describe in this work could bolster further research to elaborate the biology of skin, hair coloration, and hair health, in relevance to diet and inflammation, aging and focused on the role of glycosphingolipids. Taken together, these results demonstrate that biopolymer-encapsulated D-PDMP could be a promising agent for therapeutic use for multiple skin and hair disorders via topical use and /or by oral delivery.

## Materials and Methods

### Animal Study Approval and Protocol

All experiments were approved and followed the guideline set by the Johns Hopkins School of Medicine Animal Care and Use Committee

Male, 11-week-old apolipoprotein E-deficient (*ApoE*−/−) mice were purchased from Jackson Labs (Bar Harbor, Maine), and fed a western diet from the age of 12 to 20 weeks. Next, they were divided into the following diet/treatment groups. (A) Normal chow; (B) western diet (4.5 kcal/g, 20% fat, and 1.25% cholesterol (D12108C, Research Diet Inc, New Brunswick, NJ; (C) Western diet plus 1 mpk of biopolymer encapsulated D-PDMP (BPD); (D) western diet plus 5 mpk BPD; (E) western diet plus 10 BPD and (F) 10 mpk D-PDMP (Matreya, LLC (State College, PA)). At 20- and 36-weeks of age, mice were photographed, euthanized, and skin tissue surgically removed and store for immune-histopathological and molecular studies.

#### Measurement of the distribution of neutrophils and TSG-6 in skin tissue

Skin tissue was formalin-fixed, embedded, cut into thin sections (5-µm), and subjected to staining with hematoxylin -eosin, antibodies against a neutrophil marker (CD166), and tumor necrosis factor- alpha-induced protein (TSG-6). After staining, the skin tissue was photographed (20x). Immunochemically stained and scanned digitally, using an Asperio CS Scanscope (Vista, CA). For neutrophil- and TSG-6-positive cells, the rare event and nuclear Aperio (University of Chicago) algorithms Tool Box Kit was used. Detection was further confirmed by hand curation. Values were divided by the total cell counts of the area determined by the nuclear algorithm. TSG-6-positive mask areas were identified by dividing a TSG-6-positive mask area in (A) placebo; (B) by the area in mice treated with 5 mpk of BPD (B) and control mice skin (C). The neutrophil count and TSG-6 values in placebo, 5BP treated and control mice values were derived by counting the immunoreactive cells (brown spots) from five different areas of thin skin tissue sections from two separate mice skin specimens.

#### Quantification of skin glycosphingolipids

Sample preparation: Glycosphingolipid molecular species were extracted according to a modified Bligh and Dyer procedure^64^ Briefly, about 50-mg of each tissue sample was homogenized with 10 volumes of HPLC water at room temperature, followed by addition of 30 volumes methanol solution containing ammonium formate and d18:1/C_12:0_ ceramide as an internal standard (Avanti Polar Lipids, Alabaster, Alabama). The mixture was vortexed (Vortex-Genie, Model G560, Scientific Industries, Bohemia, NY), and 40 volumes of chloroform added. The mixture was vortexed again and centrifuged (Eppendorf centrifuge # 5424) at 1,000 *g* for 10 minutes. The chloroform (bottom layer) was separated and dried using a Savant™ SPD131 SpeedVac (Thermo Scientific, Pittsburgh, PA, USA). Dried extracts were resuspended in pure methanol and sealed and stored at -80 °C until analysis by LC-MS/MS, as described previously^62^.

#### Measurement of skin discoloration

An oval shape was drawn on the photographs taken from mice at the age of 36 weeks. This oval space covered the back area of mice from the neck to the tail (~75,000 mm^2^). Using the free drawing pen and measure/analyze tool, the white area was measured for each mouse. The ratio of white area to the total area was then calculated, and the standard deviations for all the animals in each cage (N = 5) was calculated and plotted.

#### Measurement of wounds and wound healing

For mouse skin wound analysis, the number of visible wounds in placebo- vs. BPD-treated mice (N = 5 in each group), were counted manually. Then, averages of the wounds for each group were determined, as were the ratios of wounds in BPD- vs placebo-treated mice. Those ratios were then converted to percentages, expressed as “% wound healing.”

#### Measurement of B1–4GalT-V mass in skin tissue

Here, about 10-mg skin tissue was homogenized in RIPA buffer and centrifuged at 10,000 rpm. The mass of GalT-V was measured in the supernatant fraction using an ELISA assay. About 100-µg of each protein sample (in triplicate) was loaded in 96-well, medium-binding, clear flat bottom polystyrene ELISA plates (Immulux, Chantilly VA) –total volume adjusted to 100uL with bicarbonate buffer (50 mM Na_2_CO_3_-NaHCO_3_). A synthetic GalT-V peptide having the sequence (IGAQVYEQVLRSAYAKRNSSVNDc) served as a reference standard. This antibody has been used in western immunoblot assays, ELISA assays and immunohistochemistry^65^. This antibody specifically recognizes human, mouse and rat tissue B4GalT-V. Moreover, this interaction can be inhibited by large volumes of the GalT-V peptide but not other proteins or scrambled peptide. After overnight incubation at 4 C, 200uL of blocking buffer (1%bovine serum albumin/phosphate buffered saline) was added and incubation continued overnight. Primary rabbit polyclonal antibody against GalT-V peptide was then added, and the plates incubated for 1-hr at 37 deg. C, followed by incubation for 1-hr at 37 deg. C. with a horseradish peroxidase-conjugated IgG (Sigma) secondary antibody. Finally, 100-µL of TMB solution was added, and after 10 min incubation at 37 deg. C., absorbance was measured at 450 nm.

### Analysis of gene expression by quantitative Real-Time PCR

A ~50-mg piece of skin tissue was homogenized from each mice and total RNA was isolated using TRIsol reagent according to the manufacturer’s instructions (Invitrogen, Camarillo, CA). Two microgram of RNA were reverse transcribed with SuperScript II using random primers. The primer sequence for GalT-V were as follows:

**LacCerS** CATGAACACCTCCCGATCTT, TTCATGGCCTCTTTGAAACCCCTGCCAGCTCCTTTTTCTGATG, CCTGCAGGCTTCTTCCATAG

**GlcCerS** AGTGTGTGACGGGGATGTCT, CTTCCGCAATGTACTGAGCA

**GAPDH** GGATCCACCACAGTCCATGCCATCAC, AAGCTTTCCACCACCCTGTTGCTGTA.

The primers were synthesized by Integrated DNA Technologies (Coralville, USA). Real time PCR were performed using SYBR Green PCR Master Mix PCR Master Mix (applied Biosystems, Foster City, CA, USA) in an Applied Biosystems Step one Real time PCR system as described previously^25^. Data were normalized to GAPDH mRNA levels. Expression suite software (Applied Biosystems) was used to analyze the data.

### Data availabilty

The datasets generated during and/or analyzed during the current study are available from the corresponding author on reasonable request.

### Summary

In this report, we demonstrate that inhibition of glycosphingolipid synthesis prevents numerous age-related, adverse phenotypes, related to hair biology and inflammation of the skin.

## Electronic supplementary material

Supplementary Figures

Supplementary Dataset
