# Supplementary material for: Inhibition of glycosphingolipid synthesis reverses skin inflammation and hair loss in ApoE−/− mice fed western diet
Source: Sci Rep. 2018 Jul 30;8:11463. doi: 10.1038/s41598-018-28663-9 (PMC6065400; doi:10.1038/s41598-018-28663-9)
Supplement: Supplementary file 1 — Supplementary Figures [file 41598_2018_28663_MOESM1_ESM.docx]

**Inhibition of glycosphingolipid synthesis reverses skin inflammation and hair loss in ApoE-/- mice fed western diet**

Djahida Bedja^1*^, Wenwen Yan^1, 2.*^, Viren Lad ^1^, Domenica Iocco^1^, Nickash Sivakumar^1,^ ^3^Veera Venkata Ratnam Bandaru, Subroto Chatterjee^1*^

**
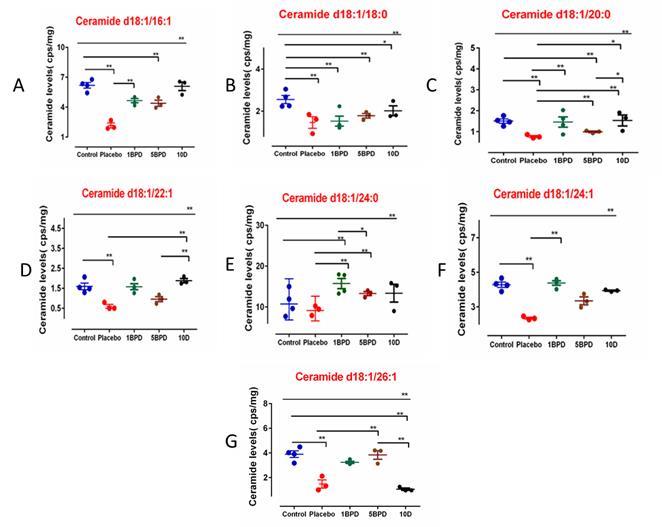
**

**Supplementary Figure 1. Level of various molecular species of ceramide in skin samples.**

**(Fig. 1S A-G)** MS/MS analysis of control mice skin ceramides showed that d18:/24:0, d18:/26:1, d18:/16:1, d18:1/24:1, d18:1/22:1, d18:1/18:0, and d18:1/20:0 were the predominant fatty acid molecular species (in the hydrophobic “tails” of ceramides), in a descending order. Western diet decreased, by ~3-fold, the levels of almost all fatty acid molecular species of ceramide, except for C18:1/24:0, which did not change significantly **(Fig. S1E)**. Treatment with 1 mpk BPD, or 10 mpk D-PDMP, on the other hand, increased them to normal levels. Neither the 10 mpk D-PDMP reverse the loss of d18:1/26:1 ceramide, nor did increasing the dose of BPD from 1 to 5 mpk, affect levels of skin ceramides, demonstrating the negative consequences of aging, including pigmentation and hair loss.

**Inhibition of glycosphingolipid synthesis reverses skin inflammation and hair loss in ApoE-/- mice fed western diet**

Djahida Bedja^1*^, Wenwen Yan^1, 2.*^, Viren Lad ^1^, Domenica Iocco^1^, Nickash Sivakumar^1,^ ^3^Veera Venkata Ratnam Bandaru, Subroto Chatterjee^1*^

**
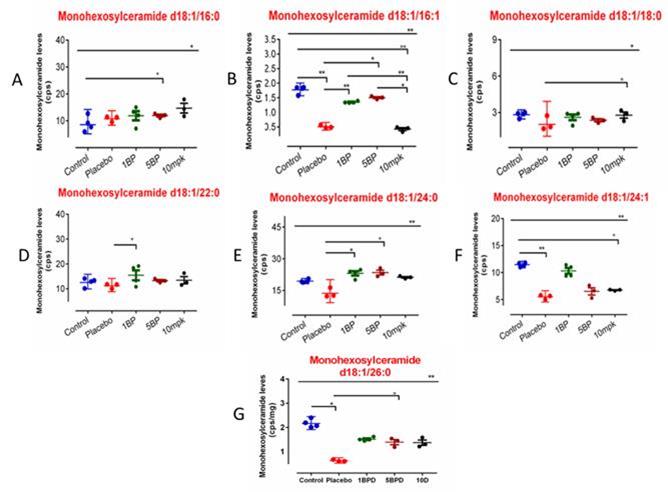
**

**Supplementary Figure 2. Level of various molecular species of monohexosyl ceramide in skin samples.** MS/MS analysis of skin monoheoxosylceramides in Apo E-/- mice fed normal chow, western diet with and without treatment with D-PDMP and BPD. A distinct, difference in the fatty acid molecular composition of skin ceramides, and their glycosylated derivatives was observed with D-PDMP treatment. The level of d18:1/16:0 monohexosylceramide was modestly increased in mice fed the western diet **(Fig. S2)**, but remained unchanged with BPD treatment. The level of monohexosylceramide containing d18:1/18:0 and d18:1/22:0 remained unchanged, with or without a western diet, or following treatment **(Fig.S 2)**. Levels of skin monohexosylceramide, having 18:1/24:0 tails, decreased by ~1.5-fold, upon western diet feeding. This level was reversed, however, upon treatment **(Fig. S2E)**, and 1 mpk D-PDMP was as efficacious as treatment with 10 mpk BPD. This treatment increased the level of d18:1/24:1, and 1 mpk BPD was superior to treatment with 10 mpk D-PDMP. The levels of skin monohexosylceramide containing d18:1/24:1 **(Fig. S2F)** and d18:1/26:0, decreased by ~ 3-fold, in mice fed a western diet. Treatment with 1 mpk BPD, but not 10 mpk D-PDMP, restored skin monohexosylceramide levels containing d18; 1/24; 1 **(Fig. S2F)** fatty acids, which were similar to monohexosylceramides containing the d18:1/26:0 fatty acid **(Fig.S 2G)**.

**Inhibition of glycosphingolipid synthesis reverses skin inflammation and hair loss in ApoE-/- mice fed western diet**

Djahida Bedja^1*^, Wenwen Yan^1, 2.*^, Viren Lad ^1^, Domenica Iocco^1^, Nickash Sivakumar^1,^ ^3^Veera Venkata Ratnam Bandaru, Subroto Chatterjee^1*^

**
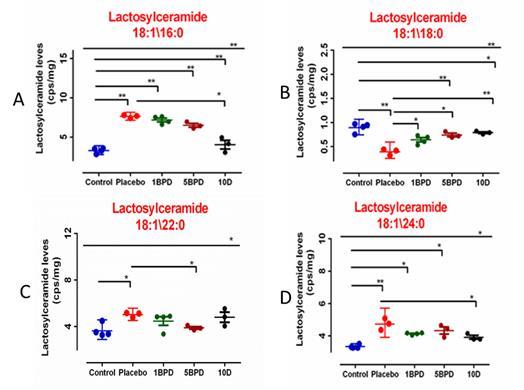
**

**Supplementary Figure 3. Level of various molecular species of lactosylceramide in skin samples.** At 36 weeks, the skin of western diet fed mice, show a 3 –fold increase in lactosylceramide having a d18:1/16:0 fatty acid, compared to control mice on a normal diet **(Fig. S3A).** D-PDMP treatment **(Fig.S3A)** moderately decreased it**.**

Contrarily, levels of lactosylceramide having d18:1/18:0 decreased ~2-fold in mice fed a western diet, compared to controls, and this was also reversed upon drug treatment **(Fig. 3B)**. Analogously, levels of lactosylceramide having d18:1/22:0 **(Fig. S3C)** and d18:1/24:1 **(Fig. S3D)** molecular species were also increased 1.5-fold and 2-fold, respectively, in the skin of mice fed a western diet. These levels were also restored to normal, upon treatment with 1 mpk BPD or 10 mpk D-PDMP **(Fig. S3C, D).**
