# Supplementary material for: Inhibition of glycosphingolipid synthesis reverses skin inflammation and hair loss in ApoE−/− mice fed western diet
Source: Sci Rep. 2018 Jul 30;8:11463. doi: 10.1038/s41598-018-28663-9 (PMC6065400; doi:10.1038/s41598-018-28663-9)
Supplement: Supplementary file 2 — Supplementary Dataset [file 41598_2018_28663_MOESM2_ESM.docx]

| **Glycolipids-skin Supplementary data** |  |  |  |  |  |
| --- | --- | --- | --- | --- | --- |
|  |  |  |  |  |  |
| Ceramide d18:1/16:1 | Control | Placebo | 1BPD | 5BPD | 10D |
| 1 | 6.192593 | 1.962406 | 5.023077 | 4.261539 | 5.242424 |
| 2 | 6.776923 | 2.684615 | 5.007813 | 4.984848 | 6.563636 |
| 3 | 5.428571 | 1.867188 | 4.42748 | 3.9375 | 6.45313 |
| 4 | 6.348837 |  | 4.166667 |  |  |
| Ceramide d18:1/18:0 | Control | Placebo | 1BPD | 5BPD | 10D |
| 1 | 2.230769 | 1.609022 | 1.353846 | 1.578125 | 1.765152 |
| 2 | 2.253846 | 0.930769 | 2.234375 | 1.8 | 2.484848 |
| 3 | 2.676923 | 1.84375 | 1.21374 | 1.962121 | 1.804688 |
| 4 | 3.045113 |  | 1.30303 |  |  |
| Ceramide d18:1/20:0 | Control | Placebo | 1BPD | 5BPD | 10D |
| 1 | 1.538462 | 0.864662 | 0.869231 | 1.007692 | 1.659091 |
| 2 | 1.769231 | 0.703846 | 2.03125 | 0.931818 | 1.037879 |
| 3 | 1.469231 | 0.753906 | 1.625954 | 1.046875 | 1.914063 |
| 4 | 1.263566 |  | 1.325758 |  |  |
| Ceramide d18:1/22:1 | Control | Placebo | 1BPD | 5BPD | 10D |
| 1 | 1.292308 | 0.501504 | 1.969231 | 0.734615 | 1.742424 |
| 2 | 1.484615 | 0.8 | 1.523438 | 0.959091 | 1.815789 |
| 3 | 2.067669 | 0.506015 | 1.267176 | 1.179688 | 2.09375 |
| 4 | 1.542636 |  | 1.567424 |  |  |
| Ceramide d18:1/24:0 | Control | Placebo | 1BPD | 5BPD | 10D |
| 1 | 15 | 10.22556 | 17.80303 | 13.46154 | 15.83333 |
| 2 | 7.692307 | 7.923077 | 13.92308 | 12.34848 | 9.015152 |
| 3 | 9.62406 | 9.453125 | 17.96875 | 14.0625 | 15.23438 |
| 4 | 11.93798 |  | 13.28244 |  |  |
| Ceramide d18:1/24:1 | Control | Placebo | 1BPD | 5BPD | 10D |
| 1 | 4.661539 | 2.458647 | 4.023077 | 3.753846 | 3.886364 |
| 2 | 4.253846 | 2.269231 | 4.609375 | 2.977273 | 3.992424 |
| 3 | 3.888889 | 2.328125 | 4.412214 | 3.320313 | 3.945313 |
| 4 | 4.333333 |  | 4.5 |  |  |
| Ceramide d18:1/26:1 | Control | Placebo | 1BPD | 5BPD | 10D |
| 1 | 4.492308 | 2.120301 | 3.1 | 3.138462 | 0.962879 |
| 2 | 3.906977 | 1.330769 | 3.195313 | 4.166667 | 1.256061 |
| 3 | 3.180451 | 1 | 3.175573 | 4.203125 | 0.969925 |
| 4 | 4.023256 |  | 3.481061 |  |  |
| Monohexosylceramide | Control | Placebo | 1BPD | 5BPD | 10D |
| d18:1/16:1-1 | 1.823077 | 0.567669 | 1.338462 | 1.553846 | 0.330303 |
| 2 | 1.853846 | 0.466154 | 1.3375 | 1.44697 | 0.487879 |
| 3 | 1.846154 | 0.485156 | 1.396183 | 1.509375 | 0.458647 |
| 4 | 1.57907 |  | 1.325758 |  |  |
| d18:1/16:0 | Control | Placebo | 1BPD | 5BPD | 10D |
| 1 | 5.915385 | 11.2782 | 13.30769 | 12.69231 | 14.42424 |
| 2 | 12.69231 | 9.538462 | 15 | 12.12121 | 17.95454 |
| 3 | 7.744361 | 11.40625 | 7.114504 | 11.01563 | 11.71875 |
| 4 | 9.147286 |  | 12.04545 |  |  |
| d18:1/18:0 | Control | Placebo | 1BPD | 5BPD | 10D |
| 1 | 2.923077 | 1.669173 | 1.923077 | 2.576923 | 3.174242 |
| 2 | 2.992308 | 1.823077 | 2.890625 | 2.143939 | 2.30303 |
| 3 | 2.488722 | 2.742188 | 2.900763 | 2.351563 | 2.875 |
| 4 | 2.887597 |  | 2.704545 |  |  |
| d18:1/22:0 | Control | Placebo | 1BPD | 5BPD | 10D |
| 1 | 13.37692 | 11.72932 | 13.30769 | 12.46154 | 16.28788 |
| 2 | 12.92308 | 10 | 19.14063 | 14.09091 | 11.51515 |
| 3 | 10.15038 | 11.82813 | 18.39695 | 13.20313 | 12.5 |
| 4 | 14.10853 |  | 10.83333 |  |  |
| d18:1/24:0 | Control | Placebo | 1BPD | 5BPD | 10D |
| 1 | 18.84615 | 16.33835 | 20.07692 | 23.07692 | 20.60606 |
| 2 | 20.61539 | 12.30769 | 24.8125 | 25.60606 | 21.22727 |
| 3 | 19.13534 | 12.65625 | 23.94657 | 21.71875 | 21.875 |
| 4 | 19.14729 |  | 23.43939 |  |  |
| d18:1/24:1 | Control | Placebo | 1BPD | 5BPD | 10D |
| 1 | 11.84615 | 5.045113 | 9.442307 | 5.292308 | 6.621212 |
| 2 | 11.69231 | 5.684615 | 11.01563 | 7.628788 | 6.848485 |
| 3 | 11.34586 | 5.796875 | 9.38168 | 6.64063 | 6.827068 |
| 4 | 11.00775 |  | 11.43182 |  |  |
| d18:1/26:0 | Control | Placebo | 1BPD | 5BPD | 10D |
| 1 | 2.007692 | 0.6 | 1.62 | 1.184615 | 1.310606 |
| 2 | 2.192308 | 0.6 | 1.492188 | 1.537879 | 1.584848 |
| 3 | 2.066165 | 0.678125 | 1.442748 | 1.445313 | 1.210938 |
| 4 | 2.4 |  | 1.500758 |  |  |
| Lactosylceramide | Control | Placebo | 1BPD | 5BPD | 10D |
| 18:1\16:0 -1 | 3.592308 | 7.804511 | 7.615385 | 6.946154 | 4.93985 |
| 2 | 2.823077 | 7.415385 | 7.53125 | 6.113636 | 3.037879 |
| 3 | 3.503759 | 7.6875 | 6.965649 | 6.429688 | 4.148438 |
| 4 | 3.255814 |  | 6.590909 |  |  |
| Lactosylceramide 18:1\18:0 | Control | Placebo | 1BPD | 5BPD | 10D |
| 1 | 0.94615 | 0.31805 | 0.60385 | 0.71769 | 0.75606 |
| 2 | 0.96899 | 0.43923 | 0.53203 | 0.69015 | 0.78788 |
| 3 | 0.90977 | 0.41641 | 0.72824 | 0.8125 | 0.82031 |
| 4 | 0.75271 |  | 0.6947 |  |  |
| 18:1\24:0 | Control | Placebo | 1BPD | 5BPD | 10D |
| 1 | 3.276923 | 4.368421 | 4.174242 | 4.430769 | 3.801515 |
| 2 | 3.276923 | 5.084615 | 4.192307 | 4.651515 | 4.151515 |
| 3 | 3.3143 | 4.757813 | 4.046875 | 3.914063 | 3.803125 |
| 4 | 3.511628 |  | 4.133588 |  |  |
| 18:1\22:0 | Control | Placebo | 1BPD | 5BPD | 10D |
| 1 | 3.369231 | 5.169173 | 4.838462 | 3.861538 | 4.054688 |
| 2 | 3.284615 | 4.784615 | 4.890625 | 4.106061 | 5.545455 |
| 3 | 3.518797 | 5.125 | 4.816794 | 3.710938 | 4.825758 |
| 4 | 4.496124 |  | 3.363636 |  |  |
| totoal -lac | Control | Placebo | 1BPD | 5BPD | 10D |
| 1 | 11.74231 | 17.30075 | 17.75154 | 13.22769 | 16.23485 |
| 2 | 11.16538 | 18.38231 | 16.78438 | 13.77348 | 16.59773 |
| 3 | 11.54887 | 18.05602 | 15.8542 | 14.10313 | 15.37578 |
| 4 | 11.4093 |  | 17.4189 | 13.59 |  |
| total-cer | Control | Placebo | 1BPD | 5BPD | 10D |
| 1 | 67.61231 | 43.30752 | 60.67652 | 54.20615 | 62.12955 |
| 2 | 54.32231 | 40.01077 | 66.69762 | 48.16439 | 58.5447 |
| 3 | 51.57444 | 42.80313 | 69.13828 | 63.50859 | 60.55078 |
| 4 | 68.02713 |  | 72.32137 |  |  |
| total-glu | Control | Placebo | 1BPD | 5BPD | 10D |
| 1 | 81.58231 | 47.43684 | 73.65692 | 63.37385 | 71.2379 |
| 2 | 83.32231 | 48.86615 | 79.06016 | 63.47297 | 75.59242 |
| 3 | 77.03233 | 46.28047 | 76.27863 | 71.67109 | 75 |
| 4 | 66.8 |  | 70.43341 |  |  |

Mouse skin wounding

TSG-6 and Neutrophil Individual and Avg count
